# Analysis of Size Correlations for Microdroplets Produced by Ultrasonic Atomization

**DOI:** 10.1155/2013/482910

**Published:** 2013-12-31

**Authors:** Annalisa Dalmoro, Anna Angela Barba, Matteo d'Amore

**Affiliations:** Dipartimento di Farmacia, Università di Salerno, Via Giovanni Paolo II 132, 84084 Fisciano (SA), Italy

## Abstract

Microencapsulation techniques are widely applied in the field of pharmaceutical production to control drugs release in time and in physiological environments. Ultrasonic-assisted atomization is a new technique to produce microencapsulated systems by a mechanical approach. Interest in this technique is due to the advantages evidenceable (low level of mechanical stress in materials, reduced energy request, reduced apparatuses size) when comparing it to more conventional techniques. In this paper, the groundwork of atomization is introduced, the role of relevant parameters in ultrasonic atomization mechanism is discussed, and correlations to predict droplets size starting from process parameters and material properties are presented and tested.

## 1. Introduction

Microencapsulation of drugs and other functional molecules is widely applied to control release rates and delivery in targeted physiological environments [1]. Microencapsulation offers greater effectiveness and microencapsulated product can be allowed lower toxicity and more lasting stability than conventional formulations [2]. Among the available microencapsulation methods, spray-drying, for preparation of microsystems, offers several advantages: it is, in principle, a continuous process, giving thus good reproducibility and potential for scale-up. The spray is usually generated by pressure and rotary nozzles that have, however, some drawbacks such as a poor control of the mean droplet size, broad droplet distributions, and risk of clogging in case of suspensions. Moreover, nozzles use only a small amount of their operating energy (either centrifugal, pressure, or kinetic) to shatter the liquid, being the greatest part transformed into kinetic energy of the particles, thus leading to large settling chambers (summarizing: costs increase when speed of the atomized particles increases) [3]. These disadvantages can be reduced using an ultrasonic atomizer: the ultrasound energy is transmitted with high efficiency to the liquid by a sonotrode, causing atomization. Although ultrasonic nozzles have not been routinely used in laboratory scale spray-drying equipment, they generate droplets, and consequently microparticles, with a uniform size distribution [4–6].

In general, the atomization is defined as the disintegration of a liquid in drops in a surrounding gas by an atomizer [5]. The resultant suspension is either defined as spray, mist, or aerosol. Atomization occurs owing to the competition between destructive and cohesive forces on the liquid surface, leading to fluctuations and disturbances in the liquid. The cohesive effect of liquid surface tension keeps the fluid in a status showing the lowest surface energy, while the stabilizing effect of viscosity tends to oppose any variation in liquid geometry. Instead, external forces, such as aerodynamic, centrifugal, and electrostatic, act on the liquid surface promoting its disintegration. The initial process of disintegration, or break-up, is defined as primary atomization. However, a number of larger droplets produced in the primary atomization can be unstable, thus reducing to smaller sizes. This process is usually defined as secondary atomization.

The effect of the forces acting on the liquid is resumed by the dimensionless numbers of Reynolds, *Re*, Weber, We, and Ohnesorge, Oh:

(1) Re=ρ·u·dpμ,We=ρ·u2·dpσ,Oh=WeRe=μρ·σ·dp,

where *ρ* is the liquid density, *u* is the liquid velocity, *d*_*p*_ is the jet diameter (primary atomization) or drop diameter (secondary atomization), *μ* is the liquid viscosity, and *σ* is the surface tension.

The Reynolds number expresses the ratio between inertial and viscous forces. The Weber number is a measure of the relative importance of fluid's inertia compared to its surface tension. By combining the two dimensionless numbers to eliminate the liquid velocity, the Ohnesorge number, containing fluid properties, is obtained. Thus, the droplets diameter can be predicted by correlations mainly based on liquid properties (density, viscosity, surface tension), on the atomizer geometry (orifice size) and on operative parameters, such as liquid flow rate. However, physical phenomena involved in the atomization processes have not been understood yet to such an extent to allow droplet size to be predicted by equations directly derivable from the first principles. The correlations proposed in the literature are mainly based on empirical studies, even if empirical correlations have been proved to be a practical way to determine droplet sizes from process parameters and relevant liquid/gas physical properties [5]. Indeed empirical correlations are very useful in the prediction of microdroplets size, especially when this feature plays a key role as it happens in pharmaceutical applications where size of drug carriers can affect rate and duration of release of the entrapped therapeutic agents.

In this study, loaded microparticles of a natural polymer which are of interest as pharmaceutical dosage forms [7] were obtained starting by ultrasonic atomization and drying processes. Fine droplets sizes were measured and calculated using predictive literature correlations. A discussion on the role of the relevant parameters in the ultrasonic atomization mechanism was also pointed out.

## 2. Materials and Methods

A biocompatible polymer, sodium alginate with high guluronic content (*FMC Bio-polymer Manugel GHB supplier*), giving more rigid gels, thus being less subjected to swelling and erosion [8, 9], was used as drug carrier for the encapsulation of a lipophilic model molecule, *α*-tocopherol vitamin (TOC) (Sigma Aldrich supplier). Also Tween 80 and calcium chloride (Sigma Aldrich supplier) were used as surfactant and as crosslinking agent, respectively.

**Figure 1:**
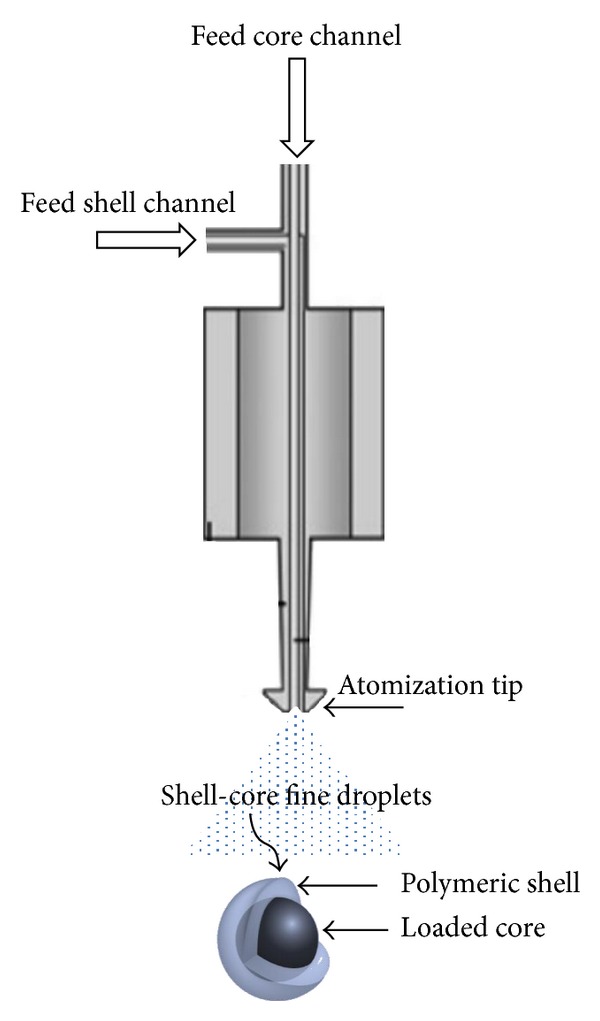
Sketch of the dual liquid feed ultrasonic atomizer probe.

**Figure 2:**
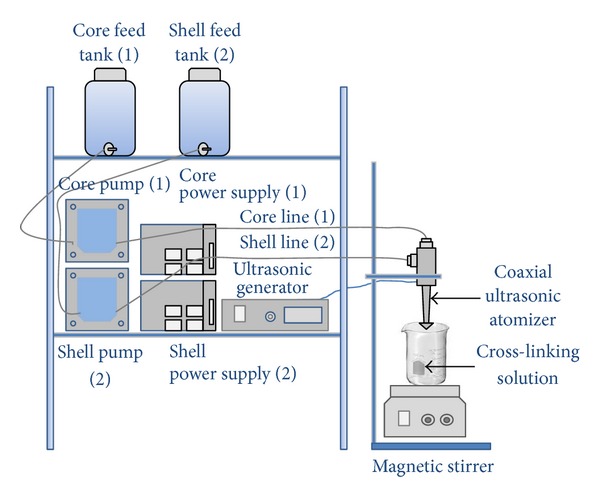
Sketch of the experimental set-up.

The ultrasonic device used to produce microparticles (Sonics & Materials Inc., CT, USA; broadband ultrasonic generator mod. 06-05108-25 KHz Sono-Tek corporation, NY, USA) was provided of a dual liquid feed probe (Sono-Tek 025-00010, Sono-Tek corporation NY, USA) (shown schematically in Figure 1). The process of particles production was already described elsewhere [10]. Briefly, the internal (*core*) and the external (*shell*) solutions (both having concentrations of alginate 1.5% w/w) were fed to the coaxial atomizer by peristaltic pumps (Verderflex OEM mod. Au EZ, RS Components Milan IT, controlled by two systems with variable tension Long WEI DC power supply PS305D to adjust the delivered solutions flow rate). In particular, the core solution was made of alginate 1.5% (w/w), *α*-tocopherol 1% (w/w), and Tween 80 0.5% (w/w). The surfactant Tween 80 was introduced in the core solution to reduce the interfacial tension and consequently the droplet dimensions; moreover, a concentration of 0.5% (w/w) was needed to obtain a stable emulsion of the water insoluble *α*-tocopherol with the water soluble solution of alginate. The two solutions came in contact only at the exit of the two channels, at the atomizer's tip, where they were nebulized, using a power of 10 W, selected to ensure an uniform distribution of the spray. The atomization products (microdroplets) were collected in a crosslinking solution at a concentration of 8.9 g/L of CaCl_2_, submitted to magnetic agitation for 5 min in a beaker. All the parameters defined for the apparatus exercise are reported in Table 1. A sketch of the experimental set-up is reported in Figure 2.

Fine particles both with matrix (obtained when only the core feed solution was pumped) and shell-core structures were obtained by the crosslinking process. They were then separated by filtration, washed, photographed (by Canon digital camera, IXUS 850 IS, by Leica digital camera DFC 280 mounted on an optical microscope, Leica Microsystems DM-LP, Wetzlar, D) and subjected to diameter measurements by image analysis as rapid and easy size characterization method (using the public domain software ImageJ 1.40 g, Wayne Rasband, National Institutes of Health, USA, freely available at http://rsb.info.nih.gov/ij/). The reticulated microdroplets were then dried by a microwave oven (De Longhi mod. Easy) until a constant weight was reached. Dried microsystems (microparticles) were thus once more subjected to diameter measurements by image analysis.

## 3. Results and Discussion

The used spray conditions (concentrations and feed rates of both internal and external solution, atomizer's frequency and power) were selected to obtain microsystems able to encapsulate the active molecule. In effect, the microsystems, as discussed in a previous work [10], showed peculiarities (high loading, delayed release) that make them of interest for specific drug delivery applications.

Image analysis for size measurements of both shell-core and matrix microparticles, done on both fresh and dried product, showed a narrow size distribution (Table 2).

Shell-core and matrix droplets (fresh microparticles) have an initial size of 78 *μ*m and 76 *μ*m, respectively. These values are in good agreement with data (related to water) of fine drop diameter distribution given by Sonotek [11]. In water atomizing by 25 kHz nozzle, 0.7% of all produced drops falls in the 10–15 *μ*m range, 1.5% falls in the subsequent 15–20 *μ*m range, and so on. The higher percentage of particles falls in a dimensional range between 70 and 80 *μ*m, as well as for alginate particles.

**Figure 3:**
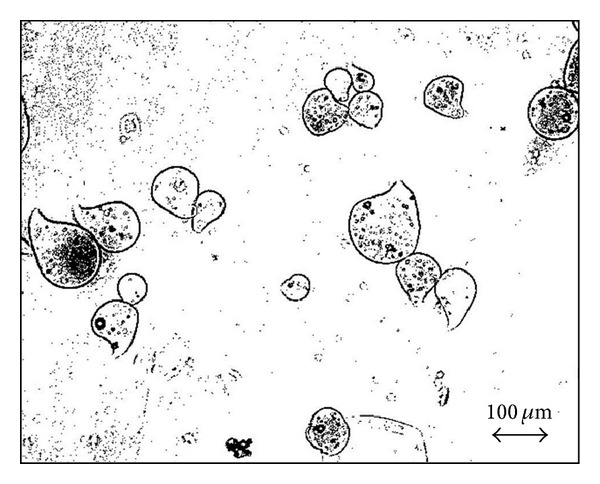
Optical microscope pictures of fresh shell-core microdroplets.

After drying, the particle size is reduced to about 40 *μ*m by a volumetric shrinkage of about 85%, as highlighted in Table 2. Moreover, fresh droplets assume a light pendant shape, as showed in Figure 3, because a spherical drop deforms due to the impact and then keeps its shape owing to the fast crosslinking [12]. This shape is essentially preserved in dried particles.

A correlation proposed for the size diameter prediction of droplets produced by ultrasonic atomization, mainly based on the frequency *f*, was given by Lang [13]:

(2) $$\begin{array}{c} d_{p}=0.34\cdot {\left(\frac{8\cdot \pi \cdot \sigma }{\rho \cdot f^{2}}\right)}^{1/3}. \end{array}$$

This correlation is only applicable when liquid phase viscosity and liquid flow rate have no effect on droplet size. However, these parameters were proven to be very important in ultrasonic atomization. The dimensionless numbers, which dictate the droplets size, were modified to consider the dependence on physical-chemical properties and ultrasonic parameters. Therefore, the concept of critical Weber, for which inertial and surface tension forces are equilibrated (We_*c*_ = 1), was extended to ultrasonic atomization, pointing out the critical flow rate, *Q*_*c*_, as the threshold above which the flow rate influences the droplets size [14]. The critical flow rate was defined as

(3) $$\begin{array}{c} Q_{c}=\frac{\sigma }{f\cdot \rho }. \end{array}$$

Then, the maximum flow rate, above which dripping takes place forming larger droplets, is considered. The maximum flow rate is the volumetric displacement rate of vibrating surface, given by the product of frequency, *f*; amplitude of sound wave, Am; and area of vibrating surface, *A*. The amplitude, Am, is defined as

(4) $$\begin{array}{c} \text{Am}=\frac{1}{2\pi \cdot f}\cdot \sqrt{\frac{2I}{\rho \cdot C}}, \end{array}$$

where *I* is the power surface intensity (defined as the ratio between the power delivered at the surface, *P*, and the area of vibrating surface, *A*) and *C* is the sound speed. The Weber number was thus modified to include the flow rate, *Q*, and the ultrasonic frequency, *f*:

(5) $$\begin{array}{c} \text{We}=\frac{f\cdot Q\cdot \rho }{\sigma }. \end{array}$$

The Ohnesorge number was also modified taking into account that in ultrasonic atomization the growth of instability is given by the amplitude, Am:

(6) $$\begin{array}{c} \text{Oh}=\frac{\mu }{f\cdot {\text{Am}}^{2}\cdot \rho }. \end{array}$$

Another dimensionless number, called intensity number, *I*_*N*_, is defined to take into account the effect of energy density on droplets size:

(7) $$\begin{array}{c} I_{N}=\frac{f^{2}\cdot {\text{Am}}^{4}}{C\cdot Q}. \end{array}$$

From these dimensionless numbers, a universal correlation was proposed by Rajan and Pandit [14]:

(8) $$\begin{array}{c} d_{p}={\left(\frac{\pi \sigma }{\rho \cdot f^{2}}\right)}^{0.33}\left[1+A\cdot {\left(\text{We}\right)}^{0.22}\cdot {\left(\text{Oh}\right)}^{0.166}\cdot {\left(I_{N}\right)}^{-0.0277}\right]. \end{array}$$

The exponents in this correlation were chosen from the experimental observations reported in the literature.

Ramisetty et al. [15] also carried out experiments and developed a correlation applicable in the following ranges: *f* = 20–130 KHz; *ρ* = 912–1151 Kg m^−3^; *σ* = 0.0029–0.073 N m^−1^; Oh = 2.71–161.64; We = 14.8–571; *I*_*N*_ = 3.65 ∗ 10^−13^–1.92 ∗ 10^−9^. The correlation was as follows:

(9) dp=0.00154·(πσρ·f2)0.33[1+(πσρ·f2)−0.2·(We)0.154           ·(Oh)−0.111·(IN)−0.033].

Avvaru et al. [16] made an attempt to include the rheological, pseudoplastic nature (non-Newtonian behaviour) of the atomizing liquid. In particular, they collected data to obtain a correlation for an aqueous solution of carboxymethylcellulose, having a shear thinning behavior, with a flow behavior index *n*:

(10) $$\begin{array}{c} d_{p}={\left(\frac{\pi \sigma }{\rho \cdot f^{2}}\right)}^{0.33}+0.0013\cdot {\left(\text{We}\right)}^{0.008}\cdot {\left(\text{Oh}\right)}^{-0.14/n}\cdot {\left(I_{N}\right)}^{0.28}. \end{array}$$

Barba et al. [12] proposed a modification of the correlation (10), applying it to the ultrasonic atomization of alginate solutions:

(11) $$\begin{array}{c} d_{p}=0.058\cdot {\left(\frac{\pi \sigma }{\rho \cdot f^{2}}\right)}^{0.33}\cdot {\left(\text{We}\right)}^{0.151}\cdot {\left(\text{Oh}\right)}^{0.192}\cdot {\left(I_{N}\right)}^{-0.02}. \end{array}$$

Therefore, the considerations enounced in the following can be done in the atomization of both Newtonian and non-Newtonian liquids.

- The droplet size decreases by increasing the frequency, *f*. The higher *f*, the larger the number of compression phases the liquid is subjected to. The crest growth is thus reduced, causing the eventual decrease of the droplets size.
- There is a range of liquid flow rate influencing the droplets size. Below a critical flow rate, *Q*_*c*_, the liquid cannot cover the whole atomizing surface and thus no effective atomization occurs. Above *Q*_*c*_, the droplets size is proportional to the liquid flow rate, again basing on film thickness on the atomization surface. Above a maximum flow rate, dripping occurs.
- An increase in ultrasonic power causes the increase of the vibration amplitude (Am), leading to a broader distribution of droplets size. At higher power, the liquid delivered on the surface immediately atomizes causing both a conical pattern of the spray and the exposition of the external part of the atomizer to air, being this latter not wetted by the liquid.
- The influence of viscosity becomes significant when it is greater than 10 cP [14]. The increase of liquid viscosity was shown to cause a reduction of droplets size. As the liquid viscosity increases, the liquid cannot be immediately atomized as it comes out from the nozzle hole. Therefore, the residence time of the liquid on the atomizing surface increases, causing liquid temperature rising, owing to the vibrational energy dissipation, and consequent decreasing of liquid viscosity to a critical value. Thus, the liquid atomizes like a low viscosity liquid giving a lower droplets size. The decrease of liquid viscosity is also enhanced by the shear thinning behavior of liquids, such as carboxymethylcellulose and alginate: the apparent viscosity on the vibrating surface decreases at the high shear rates. As a result, a pseudoplastic (non-Newtonian-shear thinning) liquid has a lower droplets size than a viscous Newtonian liquid, with a viscosity equal to the zero shear rate viscosity of the shear thinning liquid.
- When surface tension decreases, the number of capillary waves per unit of vibrating area increases at higher amplitudes, causing the immediate ejection of droplets from the crests. The higher number of droplets ejected from a liquid film at the same liquid flow rate causes a corresponding decrease in droplets size.

To summarize, a proper control of the equipment operating parameters (flow rate, frequency, and power) and liquid properties (liquid type, viscosity, and surface tension) can lead to the desired droplets size. Thus is possible to define with a good approximation of the final size of the microparticles if the effects of shrinkage are taken into account too.

The literature correlations above reported and discussed were applied to predict the size of microdroplet produced in this work. The physical properties of alginate solution at 1.5% (w/w) (Table 3) were taken by Chan et al. [17].

Since the alginate solutions have a non-Newtonian behavior, the solution viscosity can be described by the power law:

(12) $$\begin{array}{c} \eta =K\cdot {\dot{\gamma }}^{n-1}. \end{array}$$

The consistency index, *K*, and the flow index, *n*, are functions of the alginate concentration, *c*. The literature fitting equations describe this relationship in a range of alginate concentrations between 1% and 3% [12]:

(13) $$\begin{array}{c} K\left(c\right)=0.0619\cdot c^{2.953},\\ n\left(c\right)=0.9635\cdot \exp\left(-0.08\cdot c\right), \end{array}$$

where *c* is in %, *K* is in Pa · s^*n*^, and *n* is dimensionless.

The shear rate at the wall, for a power-law fluid having a volumetric flow rate *Q* flowing in a circular tube of radius *R*, is given by the following equation [12]:

(14) $$\begin{array}{c} {\dot{\gamma }}_{W}={\left[\left(\frac{1}{n}+3\right)\cdot \frac{Q}{\pi \cdot R^{3}}\right]}^{n}. \end{array}$$

For the used alginate solution, at a concentration of 1.5%, the consistency index, *K*, and the flow index, *n*, were 0.205  Pa · s^*n*^ and 0.854, respectively. The calculated shear rate at the wall, from equation (14), was 333 1/s; the consequent viscosity (from (12)) was 0.088 Pa·s. It is important to highlight some considerations:

- shell-core systems were considered homogeneous owing to the same properties of the material used for both shell and core channels and to the predominance of the shell-channel flow rate;
- actual surface tension is lower than the value reported in the literature owing to the presence of the surfactant Tween 80.

**Figure 4:**
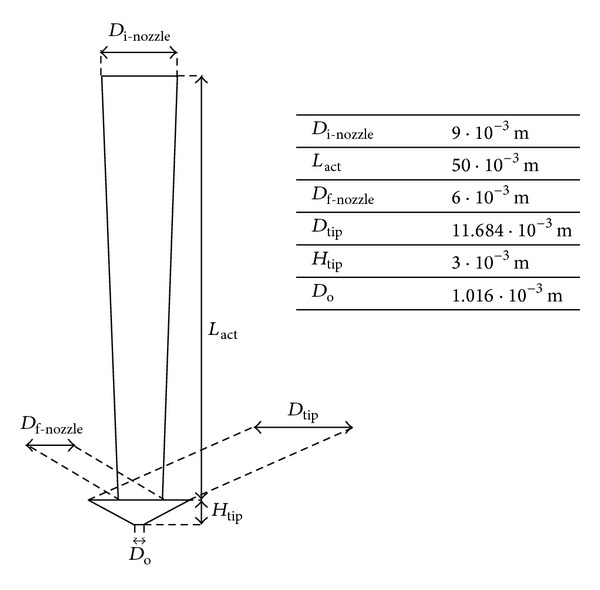
Nozzle geometry with quoted dimensions.

Liquid viscosity, atomizer geometry, and liquid flow rate have effect on the droplet size in ultrasonic atomization, as previously discussed. Actually, in the calculation of modified dimensionless numbers for ultrasonic atomization, a number of parameters related to the nozzle configuration have to be considered. Nozzle geometry and relevant dimensions are shown in Figure 4; other parameters as much as combination of some of them are in Table 4.

The dimensionless numbers assume thus the following values: We = 25.1, Oh = 8.96 · 10^3^, and *I*_*N*_ = 9.14 · 10^−13^ using (5), (6), and (7), respectively. The Weber number is larger than We_*c*_ (=1), and thus spray formation is assured. Moreover, the Ohnesorge number is very high owing to the high value of the solution viscosity (0.13 Pa·s against 0.001 Pa·s of water). Finally, both critical and maximum flow rates were evaluated to compare them with the experimental ones, that, as showed in Table 1, are 1.1 mL·min^−1^ (1.83 · 10^−8^ m^3^·s^−1^) and 4.2 mL·min^−1^ (7 · 10^−8^ m^3^·s^−1^), respectively for core and shell channels. The calculated critical flow rate was *Q*_*c*_ = 2.8 · 10^−9^ m^3^·s^−1^ with a *Q*_max⁡_ = 2.2 · 10^−5^ m^3^·s^−1^, and thus experimental flow rates are well collocated within the range of good atomization. Therefore, the three correlations previously described were applied for droplets size prediction. It is worth to note that both shell-core and matrix microdroplets showed the same size, independently from the flow rate. This phenomenon can be explained by the high viscosity of the alginate solutions (non-Newtonian shear thinning) that avoids the immediate detachment of droplet from the capillary waves formed on the surface, determining a similar droplets size distribution in a relatively small range of flow rates (in this case 1.1 and 4.2 mL·min^−1^).

Moreover, the alginate microdroplets obtained by using a higher concentration of the stabilizer Tween 80, introducing it in both shell and core material (compared to the described procedure, where Tween 80 is only in the core, thus less influencing) had a size of 54 *μ*m, lower than the size of 76–78 *μ*m previously obtained. This difference in size is due to the presence of Tween 80, which acts in two ways in the reduction of droplets diameter. First, it reduces the surface tension. Then, the presence of the stabilizer gives a higher liquid viscosity, which causes the increase of the duration of the liquid contact with the atomizing surface. The larger time of contact provokes an increase in liquid temperature and the consequent viscosity reduction. Therefore, the combined effect of reduced surface tension and reduced viscosity leads to the reduction in droplets size. However, having alginate/*α*-tocopherol a lower concentration of Tween 80, the values of proprieties of pure alginate can be used in the correlations.

The first correlation (9) proposed by Ramisetty et al. [15], giving a theoretical drop diameter of 58 *μ*m, was not able to predict the drop size because the Ohnesorge number is beyond the limits of validity of this correlation (Oh = 1.32 · 10^4^ ≫ 2.71–161.64). In effect, the alginate solution has a shear-thinning behavior, that is instead was taken into account in the correlation (10) proposed by Avvaru et al. [16]. The drop diameter predicted by the correlation (10) was of about 71 *μ*m, nearer to the measured drop size.

Furthermore, the third correlation (11), proposed by Barba et al. [12], gives a predicted particle size of 76 *μ*m, which corresponds to the value experimentally obtained, confirming the goodness of this correlation (11) tuned on alginate rather than on carboxymethylcellulose, as the previous one (10), that is anyhow a good interpretation of the phenomena occurring.

## 4. Conclusions

The fundamentals of ultrasonic atomization have been presented to point out to the basics to be used when applying the technique to the pharmaceutical field of microparticles production.

To verify the predictability of the size of the produced droplets, three empirical correlations, which take in account feed solution properties/process parameters, to predict droplets size, were used. Two correlations have been found reliable, even if at a different grade, to predict droplets size of alginate solution largely used to produce drug delivery microsystems.

## Figures and Tables

**Table 1:** Process parameters used in microdroplets production.

Parameters	Core channel	Shell channel
Feed solution features		
Flow rate, mL/min	1.1	4.2
Alginate, % w/w	1.5	1.5
*α*-tocopherol, % w/w	1	—
Tween 80, % w/w	0.5	—

Main processes time		
US atomization time: 2 min		
Crosslinking time: 5 min		

**Table 2:** Mean size and standard deviation for both shell-core and matrix microdroplets (fresh products) and microparticles (dried products) and volumetric shrinkage percentage for the dried ones.

	Mean size − SD *μ*m	Shrinkage, %
Shell-core microdroplets	78 ± 20	
Matrix microdroplets	76 ± 20	
Shell-core microparticles	41 ± 12	86
Matrix microparticles	40 ± 12	85

**Table 3:** Physical properties of *Manugel GHB* alginate solutions: the properties of a solution with a concentration of 1.5% (w/w) are in bold font.

Alginate concentration, % w/w	Density, Kg·m^−3^	Viscosity, Pa·s	Surface tension, N·m^−1^
0.5	999	0.038	0.071
**1.5**	**1004**	**0.13**	**0.07**
2.5	1008	0.56	0.069
4.0	1017	2.7	0.057
5.0	1023	4.7	0.047

**Table 4:** Values used in correlations for droplet size prediction in ultrasonic atomization.

Parametesr	Value	Notes
Vibrating surface area, *A*	1.38 · 10^−3^ m^2^	*A* is given by the sum of three areas: (1) the lateral area of a truncated cone having *D*_i-nozzle_ and *D*_f-nozzle_ as bases and *L*_act_ as height; (2) the lateral area of a truncated cone with *D*_tip_ and *D*_o_ as bases and *H*_tip_ as height; (3) the annulus area with *D*_tip_ and *D*_f-nozzle_, respectively as external and internal diameter;

Frequency, *f*	25000 Hz	

Delivered power, *P*	10 W	

Power intensity, *I*	7.25 · 10^3^ W·m^−2^	*I* = *P*/*A*

Sound speed (in water), *C*	1497 m·s^−1^	
